# Supplementary material for: Exploring the spatial working memory and visual perception in children with autism spectrum disorder and general population with high autism-like traits
Source: PLoS One. 2020 Jul 9;15(7):e0235552. doi: 10.1371/journal.pone.0235552 (PMC7347168; doi:10.1371/journal.pone.0235552)
Supplement: S1 File — (DOCX) [file pone.0235552.s002.docx]

STROBE Statement—checklist of items that should be included in reports of observational studies

|  | Item No. | Recommendation | Page  No. | Relevant text from manuscript |
| --- | --- | --- | --- | --- |
| **Title and abstract** | 1 | (*a*) Indicate the study’s design with a commonly used term in the title or the abstract | 1 | Exploring the spatial working memory and visual perception in autism children and general population with high autism-like traits |
|  |  | (*b*) Provide in the abstract an informative and balanced summary of what was done and what was found | 2 | - |
| Introduction | | | |  |
| Background/rationale | 2 | Explain the scientific background and rationale for the investigation being reported | 2 | - |
| Objectives | 3 | State specific objectives, including any prespecified hypotheses | 4 | The aim of the study is to investigate the visual perception and spatial working memory function deficits in ASD clinical individuals compared with typically developed controls. Meanwhile, we are interested in whether the results would be repeated in population study when comparing higher trait individuals with lower trait individuals. Furthermore, we detected the correlations between visual perception and spatial working memory to clarify the inner mechanisms of ASD. |
| Methods | | | |  |
| Study design | 4 | Present key elements of study design early in the paper | 4 | This study contains two parts: case-control study and community population study. |
| Setting | 5 | Describe the setting, locations, and relevant dates, including periods of recruitment, exposure, follow-up, and data collection | 4 | We recruited high-functioning ASD individuals and typically developed control children online as well as those from West China Hospital outpatient.  We also investigated in the sites of three primary schools in Pi’xian District, in the city of Chengdu, Sichuan Province, China. |
| Participants | 6 | (*a*) *Cohort study*—Give the eligibility criteria, and the sources and methods of selection of participants. Describe methods of follow-up  *Case-control study*—Give the eligibility criteria, and the sources and methods of case ascertainment and control selection. Give the rationale for the choice of cases and controls  *Cross-sectional study*—Give the eligibility criteria, and the sources and methods of selection of participants | 5 | - |
|  |  | (*b*) *Cohort study*—For matched studies, give matching criteria and number of exposed and unexposed  *Case-control study*—For matched studies, give matching criteria and the number of controls per case | 5 | - |
| Variables | 7 | Clearly define all outcomes, exposures, predictors, potential confounders, and effect modifiers. Give diagnostic criteria, if applicable | 6 |  |
| Data sources/ measurement | 8* | For each variable of interest, give sources of data and details of methods of assessment (measurement). Describe comparability of assessment methods if there is more than one group | *6* |  |
| Bias | 9 | Describe any efforts to address potential sources of bias | 6 | ANCOVA was conducted with IQ and age as covariant  The Pearson correlation analyses were conducted with age and IQ as covariant. |
| Study size | 10 | Explain how the study size was arrived at |  |  |

Continued on next page

| Quantitative variables | 11 | Explain how quantitative variables were handled in the analyses. If applicable, describe which groupings were chosen and why | 6 |  |
| --- | --- | --- | --- | --- |
| Statistical methods | 12 | (*a*) Describe all statistical methods, including those used to control for confounding | 6 | ANCOVA was conducted with IQ and age as covariant  The Pearson correlation analyses were conducted with age and IQ as covariant. |
|  |  | (*b*) Describe any methods used to examine subgroups and interactions | 6 |  |
|  |  | (*c*) Explain how missing data were addressed |  |  |
|  |  | (*d*) *Cohort study*—If applicable, explain how loss to follow-up was addressed  *Case-control study*—If applicable, explain how matching of cases and controls was addressed  *Cross-sectional study*—If applicable, describe analytical methods taking account of sampling strategy |  |  |
|  |  | (*e*) Describe any sensitivity analyses |  |  |
| Results | | | | |
| Participants | 13* | (a) Report numbers of individuals at each stage of study—eg numbers potentially eligible, examined for eligibility, confirmed eligible, included in the study, completing follow-up, and analysed | 6 | - |
|  |  | (b) Give reasons for non-participation at each stage | 4 | - |
|  |  | (c) Consider use of a flow diagram |  |  |
| Descriptive data | 14* | (a) Give characteristics of study participants (eg demographic, clinical, social) and information on exposures and potential confounders | 6 | - |
|  |  | (b) Indicate number of participants with missing data for each variable of interest |  |  |
|  |  | (c) *Cohort study*—Summarise follow-up time (eg, average and total amount) |  |  |
| Outcome data | 15* | *Cohort study*—Report numbers of outcome events or summary measures over time |  |  |
|  |  | *Case-control study—*Report numbers in each exposure category, or summary measures of exposure | *6* | *-* |
|  |  | *Cross-sectional study—*Report numbers of outcome events or summary measures | *5* | *-* |
| Main results | 16 | (*a*) Give unadjusted estimates and, if applicable, confounder-adjusted estimates and their precision (eg, 95% confidence interval). Make clear which confounders were adjusted for and why they were included | 7 | - |
|  |  | (*b*) Report category boundaries when continuous variables were categorized |  |  |
|  |  | (*c*) If relevant, consider translating estimates of relative risk into absolute risk for a meaningful time period | 8 |  |

Continued on next page

| Other analyses | 17 | Report other analyses done—eg analyses of subgroups and interactions, and sensitivity analyses |  |  |
| --- | --- | --- | --- | --- |
| Discussion | | | | |
| Key results | 18 | Summarise key results with reference to study objectives |  | 9 This study found that ASD children had abnormalities in spatial working memory, visual perception compared to typically developed controls. Furthermore, the results indicated an underneath interaction between the cognitions in the ASD group. Impairment of spatial working memory in ASD children has been demonstrated in population study, with children with high ALTs performing worse on spatial working memory than children with low ALTs. The correlation between visual perception and spatial working memory was confirmed in general population. |
| Limitations | 19 | Discuss limitations of the study, taking into account sources of potential bias or imprecision. Discuss both direction and magnitude of any potential bias |  | 11 The weakness of the study was that the gender ratio of the clinical samples was unbalanced  our study is a cross-sectional study, long-term follow-up studies can be conducted in the future, considering the impact of development on children. |
| Interpretation | 20 | Give a cautious overall interpretation of results considering objectives, limitations, multiplicity of analyses, results from similar studies, and other relevant evidence |  | 10 - |
| Generalisability | 21 | Discuss the generalisability (external validity) of the study results |  | 11 The findings of our study indicated spatial working memory played an important role in the ASD symptoms. Our results confirmed that deficits in neurocognitive functioning are autism-like traits, and have a continuous distribution in the population. The future study should be focused on the exploration of the detailed mechanisms of spatial working memory and visual perception deficits in patients with ASD. |
| Other information | |  | | |
| Funding | 22 | Give the source of funding and the role of the funders for the present study and, if applicable, for the original study on which the present article is based |  | 1 This work was supported by the National Key Research& Development Program of China (NO.2016YFC1306100) and The National Natural Science Foundation of China (NO.81371495).  We would like to thank all the families for generously donating their time so that this study could be possible. We are also grateful to the research staffs for their dedication to this project |

*Give information separately for cases and controls in case-control studies and, if applicable, for exposed and unexposed groups in cohort and cross-sectional studies.

**Note:** An Explanation and Elaboration article discusses each checklist item and gives methodological background and published examples of transparent reporting. The STROBE checklist is best used in conjunction with this article (freely available on the Web sites of PLoS Medicine at http://www.plosmedicine.org/, Annals of Internal Medicine at http://www.annals.org/, and Epidemiology at http://www.epidem.com/). Information on the STROBE Initiative is available at www.strobe-statement.org.
